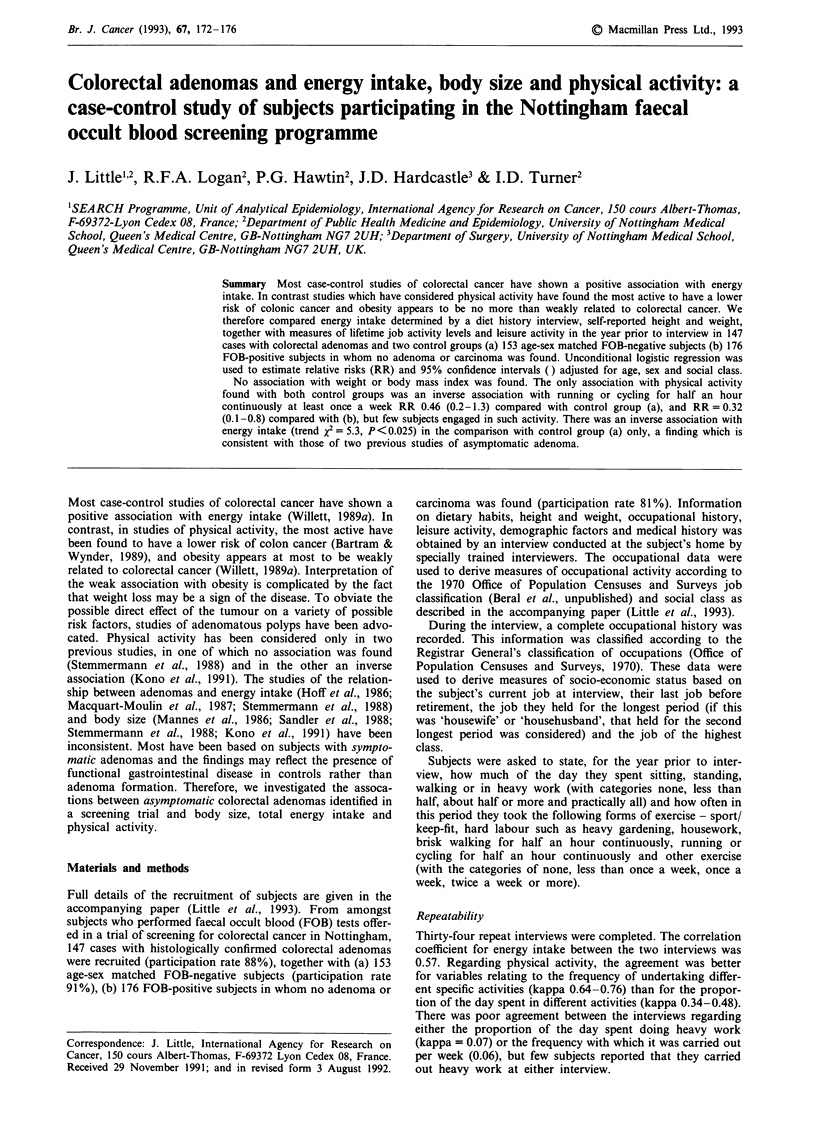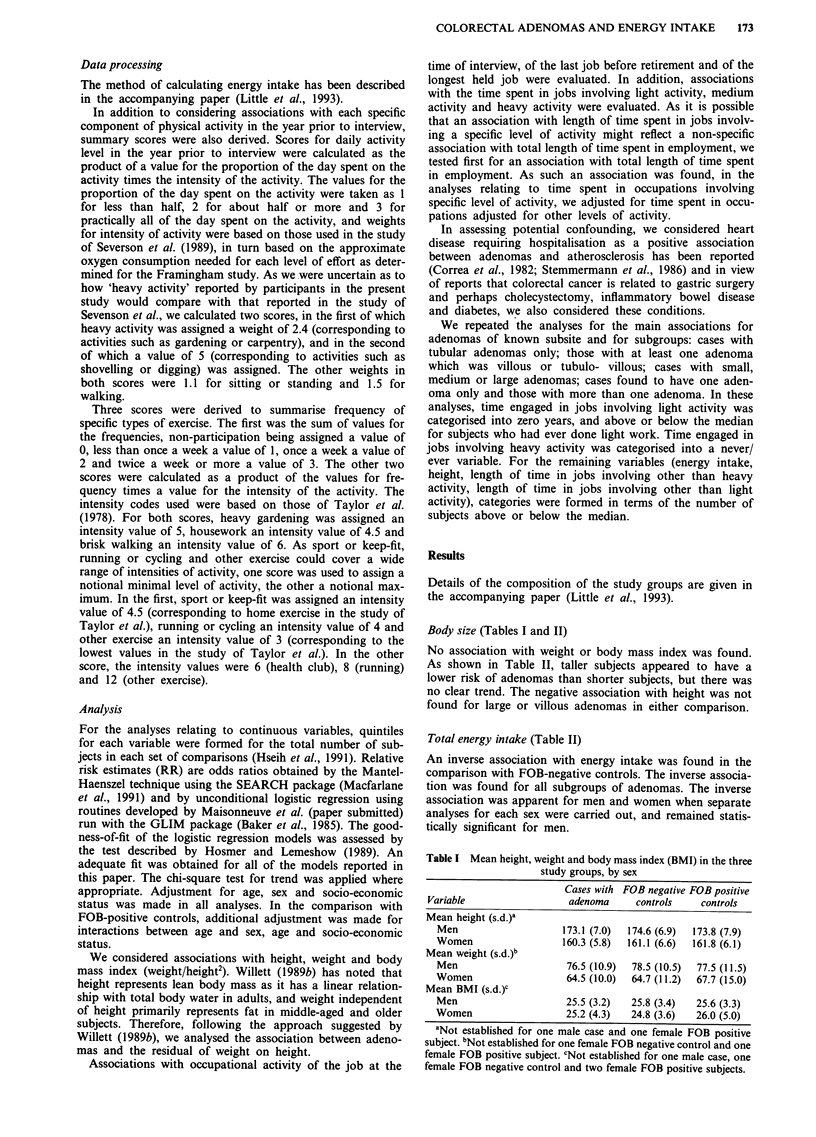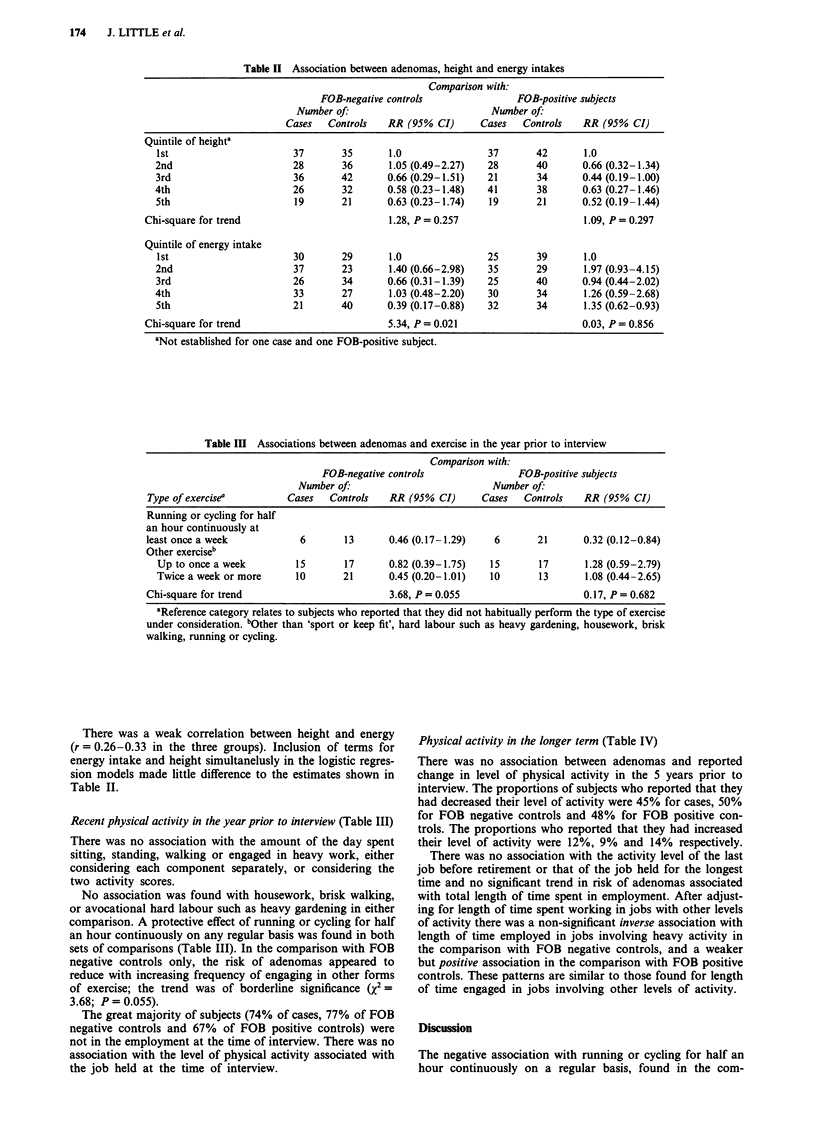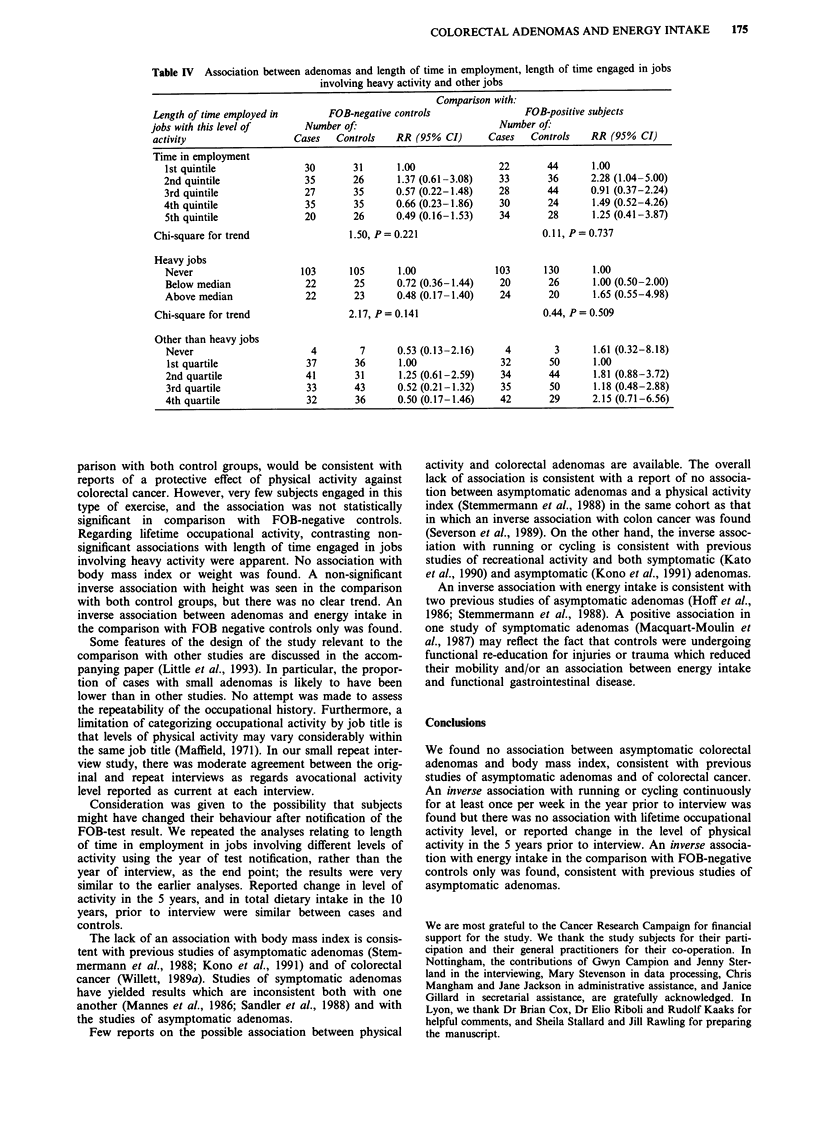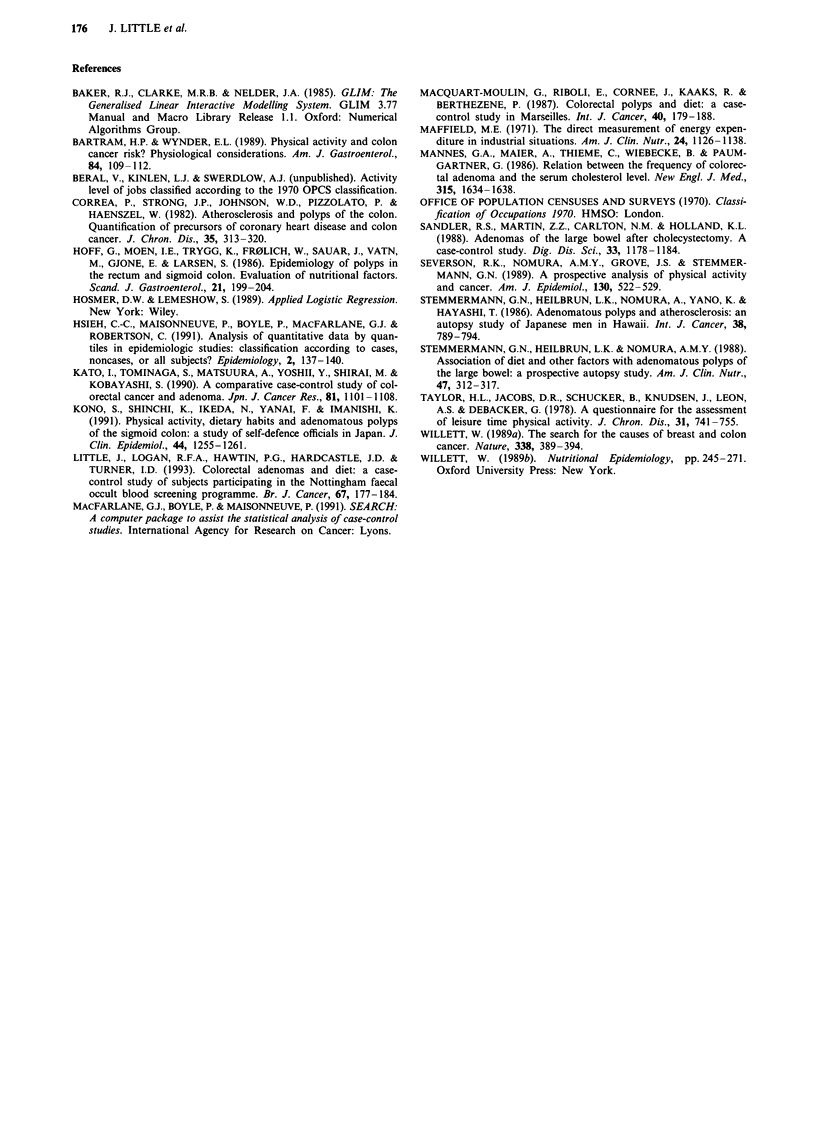# Colorectal adenomas and energy intake, body size and physical activity: a case-control study of subjects participating in the Nottingham faecal occult blood screening programme.

**DOI:** 10.1038/bjc.1993.30

**Published:** 1993-01

**Authors:** J. Little, R. F. Logan, P. G. Hawtin, J. D. Hardcastle, I. D. Turner

**Affiliations:** SEARCH Programme, Unit of Analytical Epidemiology, International Agency for Research on Cancer, Lyon, France.

## Abstract

Most case-control studies of colorectal cancer have shown a positive association with energy intake. In contrast studies which have considered physical activity have found the most active to have a lower risk of colonic cancer and obesity appears to be no more than weakly related to colorectal cancer. We therefore compared energy intake determined by a diet history interview, self-reported height and weight, together with measures of lifetime job activity levels and leisure activity in the year prior to interview in 147 cases with colorectal adenomas and two control groups (a) 153 age-sex matched FOB-negative subjects (b) 176 FOB-positive subjects in whom no adenoma or carcinoma was found. Unconditional logistic regression was used to estimate relative risks (RR) and 95% confidence intervals () adjusted for age, sex and social class. No association with weight or body mass index was found. The only association with physical activity found with both control groups was an inverse association with running or cycling for half an hour continuously at least once a week RR 0.46 (0.2-1.3) compared with control group (a), and RR = 0.32 (0.1-0.8) compared with (b), but few subjects engaged in such activity. There was an inverse association with energy intake (trend chi 2 = 5.3, P < 0.025) in the comparison with control group (a) only, a finding which is consistent with those of two previous studies of asymptomatic adenoma.


Br. J. Cancer (1993), 67, 172–176 © Macmillan Press Ltd., 1993

### Colorectal adenomas and energy intake, body size and physical activity: a case-control study of subjects participating in the Nottingham faecal occult blood screening programme

J. Little[1,2], R.F.A. Logan[2], P.G. Hawtin[2], J.D. Hardcastle[3] & I.D. Turner[2]

[1]*SEARCH Programme, Unit of Analytical Epidemiology, International Agency for Research on Cancer, 150 cours Albert-Thomas, F-69372-Lyon Cedex 08, France;* [2]*Department of Public Health Medicine and Epidemiology, University of Nottingham Medical School, Queen's Medical Centre, GB-Nottingham NG7 2UH;* [3]*Department of Surgery, University of Nottingham Medical School, Queen's Medical Centre, GB-Nottingham NG7 2UH, UK.*

**Summary** Most case-control studies of colorectal cancer have shown a positive association with energy intake. In contrast studies which have considered physical activity have found the most active to have a lower risk of colonic cancer and obesity appears to be no more than weakly related to colorectal cancer. We therefore compared energy intake determined by a diet history interview, self-reported height and weight, together with measures of lifetime job activity levels and leisure activity in the year prior to interview in 147 cases with colorectal adenomas and two control groups (a) 153 age-sex matched FOB-negative subjects (b) 176 FOB-positive subjects in whom no adenoma or carcinoma was found. Unconditional logistic regression was used to estimate relative risks (RR) and 95% confidence intervals ( ) adjusted for age, sex and social class.

No association with weight or body mass index was found. The only association with physical activity found with both control groups was an inverse association with running or cycling for half an hour continuously at least once a week RR 0.46 (0.2–1.3) compared with control group (a), and RR = 0.32 (0.1–0.8) compared with (b), but few subjects engaged in such activity. There was an inverse association with energy intake (trend $\chi^2 = 5.3$, $P < 0.025$) in the comparison with control group (a) only, a finding which is consistent with those of two previous studies of asymptomatic adenoma.

Most case-control studies of colorectal cancer have shown a positive association with energy intake (Willett, 1989*a*). In contrast, in studies of physical activity, the most active have been found to have a lower risk of colon cancer (Bartram & Wynder, 1989), and obesity appears at most to be weakly related to colorectal cancer (Willett, 1989*a*). Interpretation of the weak association with obesity is complicated by the fact that weight loss may be a sign of the disease. To obviate the possible direct effect of the tumour on a variety of possible risk factors, studies of adenomatous polyps have been advocated. Physical activity has been considered only in two previous studies, in one of which no association was found (Stemmermann *et al.*, 1988) and in the other an inverse association (Kono *et al.*, 1991). The studies of the relationship between adenomas and energy intake (Hoff *et al.*, 1986; Macquart-Moulin *et al.*, 1987; Stemmermann *et al.*, 1988) and body size (Mannes *et al.*, 1986; Sandler *et al.*, 1988; Stemmermann *et al.*, 1988; Kono *et al.*, 1991) have been inconsistent. Most have been based on subjects with *symptomatic* adenomas and the findings may reflect the presence of functional gastrointestinal disease in controls rather than adenoma formation. Therefore, we investigated the assocations between *asymptomatic* colorectal adenomas identified in a screening trial and body size, total energy intake and physical activity.

### Materials and methods

Full details of the recruitment of subjects are given in the accompanying paper (Little *et al.*, 1993). From amongst subjects who performed faecal occult blood (FOB) tests offered in a trial of screening for colorectal cancer in Nottingham, 147 cases with histologically confirmed colorectal adenomas were recruited (participation rate 88%), together with (a) 153 age-sex matched FOB-negative subjects (participation rate 91%), (b) 176 FOB-positive subjects in whom no adenoma or carcinoma was found (participation rate 81%). Information on dietary habits, height and weight, occupational history, leisure activity, demographic factors and medical history was obtained by an interview conducted at the subject's home by specially trained interviewers. The occupational data were used to derive measures of occupational activity according to the 1970 Office of Population Censuses and Surveys job classification (Beral *et al.*, unpublished) and social class as described in the accompanying paper (Little *et al.*, 1993).

During the interview, a complete occupational history was recorded. This information was classified according to the Registrar General's classification of occupations (Office of Population Censuses and Surveys, 1970). These data were used to derive measures of socio-economic status based on the subject's current job at interview, their last job before retirement, the job they held for the longest period (if this was 'housewife' or 'househusband', that held for the second longest period was considered) and the job of the highest class.

Subjects were asked to state, for the year prior to interview, how much of the day they spent sitting, standing, walking or in heavy work (with categories none, less than half, about half or more and practically all) and how often in this period they took the following forms of exercise – sport/keep-fit, hard labour such as heavy gardening, housework, brisk walking for half an hour continuously, running or cycling for half an hour continuously and other exercise (with the categories of none, less than once a week, once a week, twice a week or more).

### *Repeatability*

Thirty-four repeat interviews were completed. The correlation coefficient for energy intake between the two interviews was 0.57. Regarding physical activity, the agreement was better for variables relating to the frequency of undertaking different specific activities (kappa 0.64–0.76) than for the proportion of the day spent in different activities (kappa 0.34–0.48). There was poor agreement between the interviews regarding either the proportion of the day spent doing heavy work (kappa = 0.07) or the frequency with which it was carried out per week (0.06), but few subjects reported that they carried out heavy work at either interview.

Correspondence: J. Little, International Agency for Research on Cancer, 150 cours Albert-Thomas, F-69372 Lyon Cedex 08, France.
Received 29 November 1991; and in revised form 3 August 1992.

*Data processing*

The method of calculating energy intake has been described in the accompanying paper (Little *et al.*, 1993).

In addition to considering associations with each specific component of physical activity in the year prior to interview, summary scores were also derived. Scores for daily activity level in the year prior to interview were calculated as the product of a value for the proportion of the day spent on the activity times the intensity of the activity. The values for the proportion of the day spent on the activity were taken as 1 for less than half, 2 for about half or more and 3 for practically all of the day spent on the activity, and weights for intensity of activity were based on those used in the study of Severson *et al.* (1989), in turn based on the approximate oxygen consumption needed for each level of effort as determined for the Framingham study. As we were uncertain as to how 'heavy activity' reported by participants in the present study would compare with that reported in the study of Sevenson *et al.*, we calculated two scores, in the first of which heavy activity was assigned a weight of 2.4 (corresponding to activities such as gardening or carpentry), and in the second of which a value of 5 (corresponding to activities such as shovelling or digging) was assigned. The other weights in both scores were 1.1 for sitting or standing and 1.5 for walking.

Three scores were derived to summarise frequency of specific types of exercise. The first was the sum of values for the frequencies, non-participation being assigned a value of 0, less than once a week a value of 1, once a week a value of 2 and twice a week or more a value of 3. The other two scores were calculated as a product of the values for frequency times a value for the intensity of the activity. The intensity codes used were based on those of Taylor *et al.* (1978). For both scores, heavy gardening was assigned an intensity value of 5, housework an intensity value of 4.5 and brisk walking an intensity value of 6. As sport or keep-fit, running or cycling and other exercise could cover a wide range of intensities of activity, one score was used to assign a notional minimal level of activity, the other a notional maximum. In the first, sport or keep-fit was assigned an intensity value of 4.5 (corresponding to home exercise in the study of Taylor *et al.*), running or cycling an intensity value of 4 and other exercise an intensity value of 3 (corresponding to the lowest values in the study of Taylor *et al.*). In the other score, the intensity values were 6 (health club), 8 (running) and 12 (other exercise).

*Analysis*

For the analyses relating to continuous variables, quintiles for each variable were formed for the total number of subjects in each set of comparisons (Hseih *et al.*, 1991). Relative risk estimates (RR) are odds ratios obtained by the Mantel-Haenszel technique using the SEARCH package (Macfarlane *et al.*, 1991) and by unconditional logistic regression using routines developed by Maisonneuve *et al.* (paper submitted) run with the GLIM package (Baker *et al.*, 1985). The goodness-of-fit of the logistic regression models was assessed by the test described by Hosmer and Lemeshow (1989). An adequate fit was obtained for all of the models reported in this paper. The chi-square test for trend was applied where appropriate. Adjustment for age, sex and socio-economic status was made in all analyses. In the comparison with FOB-positive controls, additional adjustment was made for interactions between age and sex, age and socio-economic status.

We considered associations with height, weight and body mass index (weight/height$^2$). Willett (1989*b*) has noted that height represents lean body mass as it has a linear relationship with total body water in adults, and weight independent of height primarily represents fat in middle-aged and older subjects. Therefore, following the approach suggested by Willett (1989*b*), we analysed the association between adenomas and the residual of weight on height.

Associations with occupational activity of the job at the time of interview, of the last job before retirement and of the longest held job were evaluated. In addition, associations with the time spent in jobs involving light activity, medium activity and heavy activity were evaluated. As it is possible that an association with length of time spent in jobs involving a specific level of activity might reflect a non-specific association with total length of time spent in employment, we tested first for an association with total length of time spent in employment. As such an association was found, in the analyses relating to time spent in occupations involving specific level of activity, we adjusted for time spent in occupations adjusted for other levels of activity.

In assessing potential confounding, we considered heart disease requiring hospitalisation as a positive association between adenomas and atherosclerosis has been reported (Correa *et al.*, 1982; Stemmermann *et al.*, 1986) and in view of reports that colorectal cancer is related to gastric surgery and perhaps cholecystectomy, inflammatory bowel disease and diabetes, we also considered these conditions.

We repeated the analyses for the main associations for adenomas of known subsite and for subgroups: cases with tubular adenomas only; those with at least one adenoma which was villous or tubulo- villous; cases with small, medium or large adenomas; cases found to have one adenoma only and those with more than one adenoma. In these analyses, time engaged in jobs involving light activity was categorised into zero years, and above or below the median for subjects who had ever done light work. Time engaged in jobs involving heavy activity was categorised into a never/ever variable. For the remaining variables (energy intake, height, length of time in jobs involving other than heavy activity, length of time in jobs involving other than light activity), categories were formed in terms of the number of subjects above or below the median.

### Results

Details of the composition of the study groups are given in the accompanying paper (Little *et al.*, 1993).

*Body size* (Tables I and II)

No association with weight or body mass index was found. As shown in Table II, taller subjects appeared to have a lower risk of adenomas than shorter subjects, but there was no clear trend. The negative association with height was not found for large or villous adenomas in either comparison.

*Total energy intake* (Table II)

An inverse association with energy intake was found in the comparison with FOB-negative controls. The inverse association was found for all subgroups of adenomas. The inverse association was apparent for men and women when separate analyses for each sex were carried out, and remained statistically significant for men.

**Table I** Mean height, weight and body mass index (BMI) in the three study groups, by sex

| *Variable* | *Cases with adenoma* | *FOB negative controls* | *FOB positive controls* |
|---|---|---|---|
| Mean height (s.d.)[a] | | | |
| Men | 173.1 (7.0) | 174.6 (6.9) | 173.8 (7.9) |
| Women | 160.3 (5.8) | 161.1 (6.6) | 161.8 (6.1) |
| Mean weight (s.d.)[b] | | | |
| Men | 76.5 (10.9) | 78.5 (10.5) | 77.5 (11.5) |
| Women | 64.5 (10.0) | 64.7 (11.2) | 67.7 (15.0) |
| Mean BMI (s.d.)[c] | | | |
| Men | 25.5 (3.2) | 25.8 (3.4) | 25.6 (3.3) |
| Women | 25.2 (4.3) | 24.8 (3.6) | 26.0 (5.0) |

[a]Not established for one male case and one female FOB positive subject. [b]Not established for one female FOB negative control and one female FOB positive subject. [c]Not established for one male case, one female FOB negative control and two female FOB positive subjects.

**Table II** Association between adenomas, height and energy intakes

| | Comparison with: | | | | | |
|---|---|---|---|---|---|---|
| | *FOB-negative controls* | | | *FOB-positive subjects* | | |
| | *Number of:* | | | *Number of:* | | |
| | *Cases* | *Controls* | *RR (95% CI)* | *Cases* | *Controls* | *RR (95% CI)* |
| Quintile of height[a] | | | | | | |
| 1st | 37 | 35 | 1.0 | 37 | 42 | 1.0 |
| 2nd | 28 | 36 | 1.05 (0.49–2.27) | 28 | 40 | 0.66 (0.32–1.34) |
| 3rd | 36 | 42 | 0.66 (0.29–1.51) | 21 | 34 | 0.44 (0.19–1.00) |
| 4th | 26 | 32 | 0.58 (0.23–1.48) | 41 | 38 | 0.63 (0.27–1.46) |
| 5th | 19 | 21 | 0.63 (0.23–1.74) | 19 | 21 | 0.52 (0.19–1.44) |
| Chi-square for trend | | | 1.28, $P = 0.257$ | | | 1.09, $P = 0.297$ |
| Quintile of energy intake | | | | | | |
| 1st | 30 | 29 | 1.0 | 25 | 39 | 1.0 |
| 2nd | 37 | 23 | 1.40 (0.66–2.98) | 35 | 29 | 1.97 (0.93–4.15) |
| 3rd | 26 | 34 | 0.66 (0.31–1.39) | 25 | 40 | 0.94 (0.44–2.02) |
| 4th | 33 | 27 | 1.03 (0.48–2.20) | 30 | 34 | 1.26 (0.59–2.68) |
| 5th | 21 | 40 | 0.39 (0.17–0.88) | 32 | 34 | 1.35 (0.62–0.93) |
| Chi-square for trend | | | 5.34, $P = 0.021$ | | | 0.03, $P = 0.856$ |

[a]Not established for one case and one FOB-positive subject.

**Table III** Associations between adenomas and exercise in the year prior to interview

| | Comparison with: | | | | | |
|---|---|---|---|---|---|---|
| | *FOB-negative controls* | | | *FOB-positive subjects* | | |
| | *Number of:* | | | *Number of:* | | |
| *Type of exercise*[a] | *Cases* | *Controls* | *RR (95% CI)* | *Cases* | *Controls* | *RR (95% CI)* |
| Running or cycling for half an hour continuously at least once a week | 6 | 13 | 0.46 (0.17–1.29) | 6 | 21 | 0.32 (0.12–0.84) |
| Other exercise[b] | | | | | | |
| Up to once a week | 15 | 17 | 0.82 (0.39–1.75) | 15 | 17 | 1.28 (0.59–2.79) |
| Twice a week or more | 10 | 21 | 0.45 (0.20–1.01) | 10 | 13 | 1.08 (0.44–2.65) |
| Chi-square for trend | | | 3.68, $P = 0.055$ | | | 0.17, $P = 0.682$ |

[a]Reference category relates to subjects who reported that they did not habitually perform the type of exercise under consideration. [b]Other than 'sport or keep fit', hard labour such as heavy gardening, housework, brisk walking, running or cycling.

There was a weak correlation between height and energy ($r = 0.26–0.33$ in the three groups). Inclusion of terms for energy intake and height simultanelusly in the logistic regression models made little difference to the estimates shown in Table II.

*Recent physical activity in the year prior to interview* (Table III)

There was no association with the amount of the day spent sitting, standing, walking or engaged in heavy work, either considering each component separately, or considering the two activity scores.

No association was found with housework, brisk walking, or avocational hard labour such as heavy gardening in either comparison. A protective effect of running or cycling for half an hour continuously on any regular basis was found in both sets of comparisons (Table III). In the comparison with FOB negative controls only, the risk of adenomas appeared to reduce with increasing frequency of engaging in other forms of exercise; the trend was of borderline significance ($\chi^2 = 3.68$; $P = 0.055$).

The great majority of subjects (74% of cases, 77% of FOB negative controls and 67% of FOB positive controls) were not in the employment at the time of interview. There was no association with the level of physical activity associated with the job held at the time of interview.

*Physical activity in the longer term* (Table IV)

There was no association between adenomas and reported change in level of physical activity in the 5 years prior to interview. The proportions of subjects who reported that they had decreased their level of activity were 45% for cases, 50% for FOB negative controls and 48% for FOB positive controls. The proportions who reported that they had increased their level of activity were 12%, 9% and 14% respectively.

There was no association with the activity level of the last job before retirement or that of the job held for the longest time and no significant trend in risk of adenomas associated with total length of time spent in employment. After adjusting for length of time spent working in jobs with other levels of activity there was a non-significant *inverse* association with length of time employed in jobs involving heavy activity in the comparison with FOB negative controls, and a weaker but *positive* association in the comparison with FOB positive controls. These patterns are similar to those found for length of time engaged in jobs involving other levels of activity.

### Discussion

The negative association with running or cycling for half an hour continuously on a regular basis, found in the com-

**Table IV** Association between adenomas and length of time in employment, length of time engaged in jobs involving heavy activity and other jobs

| Length of time employed in jobs with this level of activity | Comparison with: FOB-negative controls Number of: Cases | Controls | RR (95% CI) | FOB-positive subjects Number of: Cases | Controls | RR (95% CI) |
|---|---|---|---|---|---|---|
| Time in employment | | | | | | |
| 1st quintile | 30 | 31 | 1.00 | 22 | 44 | 1.00 |
| 2nd quintile | 35 | 26 | 1.37 (0.61–3.08) | 33 | 36 | 2.28 (1.04–5.00) |
| 3rd quintile | 27 | 35 | 0.57 (0.22–1.48) | 28 | 44 | 0.91 (0.37–2.24) |
| 4th quintile | 35 | 35 | 0.66 (0.23–1.86) | 30 | 24 | 1.49 (0.52–4.26) |
| 5th quintile | 20 | 26 | 0.49 (0.16–1.53) | 34 | 28 | 1.25 (0.41–3.87) |
| Chi-square for trend | | 1.50, $P = 0.221$ | | | 0.11, $P = 0.737$ | |
| Heavy jobs | | | | | | |
| Never | 103 | 105 | 1.00 | 103 | 130 | 1.00 |
| Below median | 22 | 25 | 0.72 (0.36–1.44) | 20 | 26 | 1.00 (0.50–2.00) |
| Above median | 22 | 23 | 0.48 (0.17–1.40) | 24 | 20 | 1.65 (0.55–4.98) |
| Chi-square for trend | | 2.17, $P = 0.141$ | | | 0.44, $P = 0.509$ | |
| Other than heavy jobs | | | | | | |
| Never | 4 | 7 | 0.53 (0.13–2.16) | 4 | 3 | 1.61 (0.32–8.18) |
| 1st quartile | 37 | 36 | 1.00 | 32 | 50 | 1.00 |
| 2nd quartile | 41 | 31 | 1.25 (0.61–2.59) | 34 | 44 | 1.81 (0.88–3.72) |
| 3rd quartile | 33 | 43 | 0.52 (0.21–1.32) | 35 | 50 | 1.18 (0.48–2.88) |
| 4th quartile | 32 | 36 | 0.50 (0.17–1.46) | 42 | 29 | 2.15 (0.71–6.56) |

parison with both control groups, would be consistent with reports of a protective effect of physical activity against colorectal cancer. However, very few subjects engaged in this type of exercise, and the association was not statistically significant in comparison with FOB-negative controls. Regarding lifetime occupational activity, contrasting non-significant associations with length of time engaged in jobs involving heavy activity were apparent. No association with body mass index or weight was found. A non-significant inverse association with height was seen in the comparison with both control groups, but there was no clear trend. An inverse association between adenomas and energy intake in the comparison with FOB negative controls only was found.

Some features of the design of the study relevant to the comparison with other studies are discussed in the accompanying paper (Little *et al.*, 1993). In particular, the proportion of cases with small adenomas is likely to have been lower than in other studies. No attempt was made to assess the repeatability of the occupational history. Furthermore, a limitation of categorizing occupational activity by job title is that levels of physical activity may vary considerably within the same job title (Maffield, 1971). In our small repeat interview study, there was moderate agreement between the original and repeat interviews as regards avocational activity level reported as current at each interview.

Consideration was given to the possibility that subjects might have changed their behaviour after notification of the FOB-test result. We repeated the analyses relating to length of time in employment in jobs involving different levels of activity using the year of test notification, rather than the year of interview, as the end point; the results were very similar to the earlier analyses. Reported change in level of activity in the 5 years, and in total dietary intake in the 10 years, prior to interview were similar between cases and controls.

The lack of an association with body mass index is consistent with previous studies of asymptomatic adenomas (Stemmermann *et al.*, 1988; Kono *et al.*, 1991) and of colorectal cancer (Willett, 1989*a*). Studies of symptomatic adenomas have yielded results which are inconsistent both with one another (Mannes *et al.*, 1986; Sandler *et al.*, 1988) and with the studies of asymptomatic adenomas.

Few reports on the possible association between physical activity and colorectal adenomas are available. The overall lack of association is consistent with a report of no association between asymptomatic adenomas and a physical activity index (Stemmermann *et al.*, 1988) in the same cohort as that in which an inverse association with colon cancer was found (Severson *et al.*, 1989). On the other hand, the inverse association with running or cycling is consistent with previous studies of recreational activity and both symptomatic (Kato *et al.*, 1990) and asymptomatic (Kono *et al.*, 1991) adenomas.

An inverse association with energy intake is consistent with two previous studies of asymptomatic adenomas (Hoff *et al.*, 1986; Stemmermann *et al.*, 1988). A positive association in one study of symptomatic adenomas (Macquart-Moulin *et al.*, 1987) may reflect the fact that controls were undergoing functional re-education for injuries or trauma which reduced their mobility and/or an association between energy intake and functional gastrointestinal disease.

### Conclusions

We found no association between asymptomatic colorectal adenomas and body mass index, consistent with previous studies of asymptomatic adenomas and of colorectal cancer. An *inverse* association with running or cycling continuously for at least once per week in the year prior to interview was found but there was no association with lifetime occupational activity level, or reported change in the level of physical activity in the 5 years prior to interview. An *inverse* association with energy intake in the comparison with FOB-negative controls only was found, consistent with previous studies of asymptomatic adenomas.

We are most grateful to the Cancer Research Campaign for financial support for the study. We thank the study subjects for their participation and their general practitioners for their co-operation. In Nottingham, the contributions of Gwyn Campion and Jenny Sterland in the interviewing, Mary Stevenson in data processing, Chris Mangham and Jane Jackson in administrative assistance, and Janice Gillard in secretarial assistance, are gratefully acknowledged. In Lyon, we thank Dr Brian Cox, Dr Elio Riboli and Rudolf Kaaks for helpful comments, and Sheila Stallard and Jill Rawling for preparing the manuscript.

### References

BAKER, R.J., CLARKE, M.R.B. & NELDER, J.A. (1985). *GLIM: The Generalised Linear Interactive Modelling System*. GLIM 3.77 Manual and Macro Library Release 1.1. Oxford: Numerical Algorithms Group.

BARTRAM, H.P. & WYNDER, E.L. (1989). Physical activity and colon cancer risk? Physiological considerations. *Am. J. Gastroenterol.*, **84**, 109–112.

BERAL, V., KINLEN, L.J. & SWERDLOW, A.J. (unpublished). Activity level of jobs classified according to the 1970 OPCS classification.

CORREA, P., STRONG, J.P., JOHNSON, W.D., PIZZOLATO, P. & HAENSZEL, W. (1982). Atherosclerosis and polyps of the colon. Quantification of precursors of coronary heart disease and colon cancer. *J. Chron. Dis.*, **35**, 313–320.

HOFF, G., MOEN, I.E., TRYGG, K., FRØLICH, W., SAUAR, J., VATN, M., GJONE, E. & LARSEN, S. (1986). Epidemiology of polyps in the rectum and sigmoid colon. Evaluation of nutritional factors. *Scand. J. Gastroenterol.*, **21**, 199–204.

HOSMER, D.W. & LEMESHOW, S. (1989). *Applied Logistic Regression*. New York: Wiley.

HSIEH, C.-C., MAISONNEUVE, P., BOYLE, P., MACFARLANE, G.J. & ROBERTSON, C. (1991). Analysis of quantitative data by quantiles in epidemiologic studies: classification according to cases, noncases, or all subjects? *Epidemiology*, **2**, 137–140.

KATO, I., TOMINAGA, S., MATSUURA, A., YOSHII, Y., SHIRAI, M. & KOBAYASHI, S. (1990). A comparative case-control study of colorectal cancer and adenoma. *Jpn. J. Cancer Res.*, **81**, 1101–1108.

KONO, S., SHINCHI, K., IKEDA, N., YANAI, F. & IMANISHI, K. (1991). Physical activity, dietary habits and adenomatous polyps of the sigmoid colon: a study of self-defence officials in Japan. *J. Clin. Epidemiol.*, **44**, 1255–1261.

LITTLE, J., LOGAN, R.F.A., HAWTIN, P.G., HARDCASTLE, J.D. & TURNER, I.D. (1993). Colorectal adenomas and diet: a case-control study of subjects participating in the Nottingham faecal occult blood screening programme. *Br. J. Cancer*, **67**, 177–184.

MACFARLANE, G.J., BOYLE, P. & MAISONNEUVE, P. (1991). *SEARCH: A computer package to assist the statistical analysis of case-control studies*. International Agency for Research on Cancer: Lyons.

MACQUART-MOULIN, G., RIBOLI, E., CORNEE, J., KAAKS, R. & BERTHEZENE, P. (1987). Colorectal polyps and diet: a case-control study in Marseilles. *Int. J. Cancer*, **40**, 179–188.

MAFFIELD, M.E. (1971). The direct measurement of energy expenditure in industrial situations. *Am. J. Clin. Nutr.*, **24**, 1126–1138.

MANNES, G.A., MAIER, A., THIEME, C., WIEBECKE, B. & PAUMGARTNER, G. (1986). Relation between the frequency of colorectal adenoma and the serum cholesterol level. *New Engl. J. Med.*, **315**, 1634–1638.

OFFICE OF POPULATION CENSUSES AND SURVEYS (1970). *Classification of Occupations 1970*. HMSO: London.

SANDLER, R.S., MARTIN, Z.Z., CARLTON, N.M. & HOLLAND, K.L. (1988). Adenomas of the large bowel after cholecystectomy. A case-control study. *Dig. Dis. Sci.*, **33**, 1178–1184.

SEVERSON, R.K., NOMURA, A.M.Y., GROVE, J.S. & STEMMERMANN, G.N. (1989). A prospective analysis of physical activity and cancer. *Am. J. Epidemiol.*, **130**, 522–529.

STEMMERMANN, G.N., HEILBRUN, L.K., NOMURA, A., YANO, K. & HAYASHI, T. (1986). Adenomatous polyps and atherosclerosis: an autopsy study of Japanese men in Hawaii. *Int. J. Cancer*, **38**, 789–794.

STEMMERMANN, G.N., HEILBRUN, L.K. & NOMURA, A.M.Y. (1988). Association of diet and other factors with adenomatous polyps of the large bowel: a prospective autopsy study. *Am. J. Clin. Nutr.*, **47**, 312–317.

TAYLOR, H.L., JACOBS, D.R., SCHUCKER, B., KNUDSEN, J., LEON, A.S. & DEBACKER, G. (1978). A questionnaire for the assessment of leisure time physical activity. *J. Chron. Dis.*, **31**, 741–755.

WILLETT, W. (1989*a*). The search for the causes of breast and colon cancer. *Nature*, **338**, 389–394.

WILLETT, W. (1989*b*). *Nutritional Epidemiology*, pp. 245–271. Oxford University Press: New York.